# Empirical Investigation of Work-Related Social Media Usage and Social-Related Social Media Usage on Employees’ Work Performance

**DOI:** 10.3390/bs12080297

**Published:** 2022-08-20

**Authors:** Rui Miguel Dantas, Hira Aftab, Sumaira Aslam, Muhammad Ussama Majeed, Anabela Batista Correia, Hamza Ahmad Qureshi, João Luis Lucas

**Affiliations:** 1 ISCAL (Instituto Superior de Contabilidade e Administração de Lisboa), Instituto Politécnico de Lisboa, Avenida Miguel Bombarda, 20, 1069-035 Lisboa, Portugal; 2 Institute of Business and Information Technology, University of the Punjab, Lahore 54590, Pakistan; 3 School of Commerce and Accountancy, University of Management and Technology, Lahore 54000, Pakistan; 4 Department of Management Sciences, National University of Modern Languages, Lahore Campus, Lahore 54000, Pakistan or; 5 Departamento de Gestão, Universidade de Évora, Largo Dos Colegiais, 2, 7002-554 Évora, Portugal

**Keywords:** work-related social media, work performance, extra-role behavior, socializing, social media at the workplace, personal usage of social media

## Abstract

The trend of using social media in the workplace is now becoming ubiquitous. Along witbenefits, social media also has negative consequences. Employees use social media for both work and social purposes. Therefore, using a quantitative approach, this study explores the impact of work-related social media usage and social-related social media usage on employees’ work performance. This study also investigates the mediating effect of extra-role behaviors on social media usage (professional and personal purpose) and work performance relationships. We examined survey data of 241 employees working in different organizations with the help of the partial least square (PLS) 3.0 version. Convenience sampling has been used to reach respondents. The outcomes of this study demonstrate that both professional and personal-related social media usage have a positive and significant impact on employees’ work performance. This study also highlighted that extra-role behavior positively and significantly mediates the relationship between social media usage (work and personal) and employees’ work performance. This study provides practical insights to managers, such as that, instead of banning social media usage in companies, there must be some limits and regulations for using social media that would facilitate firms to increase employees’ engagement and productivity.

## 1. Introduction

Social media is commonly utilized as an umbrella term that depicts various online platforms, including blogs, business networks, collaborative projects, enterprise social networks, forum blogging, photo sharing, video sharing, social bookmarking, and the virtual world. From this wide range of social media stages, the utilization of social media is quite diverse and not limited to advertising and promotions [1]. The social media platform has remodeled the world through the communication and dissemination of information. The use of social media for both personal and work purposes has a significant effect on human personality.

Social media networking sites have been classified into two main categories: publicly used social networking sites and internal social networking sites. Public social networking sites are for the everyday use of the public by commercial providers, and they are mostly free. Typical examples are Facebook, Twitter, LinkedIn, My Space, and Friendster. Organizations run internal social networking sites on their server specifically for their internal use. Examples of this include Water cooler by HP, Town Square by Microsoft, Harmony by SAP, D Street by Deloitte, and a beehive by IBM [2].

Due to the complexity of jobs in the present day, it is an unavoidable fact that no one can work alone; thus, collaboration is required to get things done. Hence, it has become a rapidly acceptable practice for employees to use social media internally and externally to achieve organizational outcomes. Social media has a significant effect on business and private life by changing the nature and efficiency of the communication process [3].

Research on social media in organizations has predominantly focused on two essential perspectives. The first perspective is related to investigating the factors influencing social media usage, mainly including the technology, organizational, environmental, and personal factors. The second perspective explores the direct impact of social media usage on job satisfaction and job performance. However, research regarding different aspects of social media usage is still in the initial phase; this is why only a few researchers have examined both aspects of social media usage, namely work-related social media usage and social-related social media usage. Work-related social media usage is also termed as professional use of social media. Similarly, social-related social media usage is also known as personal usage of social media.

In today’s challenging world, organizations need to construct a productive and sustainable environment. Employees must be prepared to do more than the minimum and recognize the specific technical elements of their job. To increase an organization’s performance, an employee should actively participate in optional and non-obligatory work, such as extra-role behaviors. The term extra-role behavior refers to the course of actions or conduct of the employees that are not part of the formal job description of the employee. Such behavior paves the way for an organization to operate smoothly as a social framework [4].

Generally, the research inspecting the effect of extra-role behaviors on organizational performance is limited. Therefore, there is a need to understand the apparent relationship of extra-role behaviors with work performance while considering the two main motivations of social media usage [5]. Social media usage help firms in knowledge management, information sharing, connecting with customers, providing better services to clients all over the world, and in helping in the promotion of the firm [6,7]. Nevertheless, many firms do not allow their employees to spend time on social media at the workplace to avoid time wastage and concentration divergence [8].

The employees’ social media usage at their workplace is an ongoing debate. It is of significant concern for the employees as they fear that such practice can lower their productivity, leading them to unemployment. Restrictions are imposed in organizations to lessen the use of social media at the workplace because higher authorities believe that social media usage can decrease the level of employee commitment to the job and the organization. Therefore, investigating whether social media usage affects the work performance of employees is essential. The present study explores social media usage for both work and social purposes on work performance. This study also clarifies the relationship of extra-role behavior with social media usage and work performance and reveals its path process by investigating the mediating effect of extra-role behaviors on the social media usage (work and social)–work performance relationship.

Based on these discussions, the research questions (RQs) of the study are as follows:

1. Does work-related social media usage impact the employees’ work performance?
2. Does personal use of social media impact the employees’ work performance?
3. Do extra-role behaviors mediate the relationship between work-related social media usage and employees’ work performance?
4. Do extra-role behaviors mediate the relationship between social-related social media usage and employees’ work performance?

### Literature Review

Social medias are web-based services that allow individuals, communities, and organizations to collaborate, connect, interact, and build a community by enabling them to create, co-create, modify, share, and engage with user-generated content that is easily accessible [9]. Social media has become an integral element of everyday life. Social media fosters open communication, which improves the collection and transmission of information. It also allows employees to exchange resources, discuss ideas, publish news, and ask questions. Additionally, social media allows for expanding relationships and partnerships. These digital platforms provide the ability to boost worker productivity by connecting employees to global resources [10]. Nowadays, social media is used for obtaining technical help and expressing ideas at work [11]. The most positive aspect of social media used for work-related is that all the organization’s employees share their experiences on social media [12].

Moreover, social media users disclose their workplace and environment with the help of social media status or posts. This publicly available data is also a rich source of information for employers as it is critical feedback from the employees [13]. Social technologies can provide a reliable way for organizations to deal with an information flow that initiates the change in the knowledge management system linked to performance [14].

Workplace relationships across personal and professional boundaries are expanded by utilizing social technologies represented as social networking sites [15]. Another study by Taha (2018) explored how employees use social media networks to communicate with family, companions, and different organizations in the working environment, and this is a source of retaining employees, especially fresh recruits [16]. Social networking sites and applications can be used proficiently to cultivate information-sharing practice in the work environment. Knowledge-sharing culture fosters effective communication, and employee involvement can be enhanced by using social media networking tools [17]. In another research Holland and his colleagues (2019) highlighted the two benefits of social media usage: Firstly, employees can discuss difficulties faced due to the management at the workplace. The second is that building internal social media at the workplace can connect an employee’s voice on the workplace issues with the management [18].

Organizational strategies such as information dissemination and two-way symmetrical communication encourage internal social media usage [19]. Another study revealed the barriers and drivers of knowledge sharing within the organization. Enjoyment in helping others, monetary rewards, management support, management encouragement, and knowledge-sharing behavior we reconsidered the significant drivers of knowledge sharing using social media. In contrast, a lack of trust and change of conduct was regarded as risk [20]. Enterprise social media is an emerging technology that organizations are embracing as a digital platform that encourages communication between employees. There is a positive relationship between enterprise social media usage and employee agility [21].

Social networking sites fulfill numerous needs, including networking, communication, recruitment, and sharing knowledge. Social media has five named components: clients’ interest and skills, graphic skills, organizational support, and availability of equipment and the internet. These five components significantly affect the influence of social media and attitudes toward social media [22]. Addiction to social media has increased at the workplace. The internet is turning into a significant source for online users to look for content or social gratification. If there is no proper control over the use of the internet, it can exacerbate various problems such as depression, sexual disorders, or loneliness in the workplace [23]. Social media use can be classified into two main categories, namely for personal and work-related purposes. Personal use of social media means using it for enjoyment, playing games, setting social events with co-workers, and other personal activities online. Work-related use of social media may be defined as indulging oneself in online work-related posts, content sharing, discussions, tweeting about the work-related experiences, and communication at the workplace. This use ultimately impacts the extra role behavior of the employees at the workplace [24].

Cyber slacking is a term used to define social media usage for personal purposes at the workplace. Cyber loafing is also referred to as the behavior of the employees using the internet provided by the organization for personal or non-job purposes at the workplace [25,26]. Approximately, employees spend at least 1 h on non-work-related activities, and the most considerable portion of these activities was on the internet. This issue has become a concern for employers. Employers are concerned because they think that social media usage during working hours leads to reduced productivity, leading to financial loss [27].

When people are stressed and averse to their surroundings, they engage in cyber-slacking as a way to escape [28]. Individuals that engage in cyber slacking do so to relieve stress and to find enjoyment, both of which are important for psychological well-being [29]. Gibson (1977) established the concept of “affordance theory,” which is based on the idea that an individual’s perceptions drive their actions. His insightful study of the interactions between people or animals and their living situations is particularly noteworthy. Gibson claimed that an animal or a person observes a thing not for what it is, but for what it might be or what kinds of applications it can accommodate [30]. The term “affordances” is used in the study to refer to the usefulness of anything. Since Gibson (1977) introduced the concept of affordance, researchers have used the affordance lens to investigate the relationship between social interaction driven by technology advancements in its dynamic [31] and how new technology may be implemented and improved in terms of design [32]. Aside from that, another study provided some valuable insights into the corpus of human–computer interaction (HCI) research for practitioners. First and foremost, the findings of that study imply that social connections among workers may be used as a means of reducing cyber slacking in the workplace [33]. Employees should be given the opportunity to participate in two-way conversations with their managers. For example, to encourage social connection among workers, a manager can consider arranging corporate leisure activities such as eating together, athletic activities, and departmental travel [34].

The activities included under the work-related usage of social media are creating groups or communities to make connections and interaction with the clients or customers. Similarly, crowd sourcing through social media can generate innovative ideas for products and services. Moreover, widespread marketing programs for creating support community at work, searching for job applicants, searching for job vacancies, starting a business network, acting as a sales channel, and content sharing are considered to be the main aspects of work-related social media usage. It has become challenging for organizations to control and check on their employees’ activities during working hours at the workplace due to the technology-driven environment and the widespread social media usage [35].

Job-related stress harms the employees’ productivity, and it is also disturbing the employees’ social life. Work-related stress is also a major cause of family imbalance issues in Pakistan [36,37]. To reduce job-related stress, employers are more concerned about the workplace environment and effective communication of employees. Recent research suggests that the use of social media for the communication of employees with their families reduces job-related stress at the workplace [38].

Along with the power of social media and the risk associated with it, employees’ level of expectation is changing, and they feel comfortable using these social sites [39]. The notion that employees can do their work online is the other primary justification given for the use of social media while at work. The additional logic that is given to using social media for personal tasks is that employees will be happier and more productive if they spend their time on social media for socializing with people online during working hours [40]. It is a fact that the chance of irregular activity by employees increases by allowing the use of social media for social service. Still, it can be more detrimental for the organization if they completely curb social media for personal use, as it may lead to a lack of engagement and productivity at the workplace [41]. Organizations may start recognizing the value of social media use by the employees at the workplace if making a social connection and relations are the driver of a contented workplace. This would ultimately lead to increased job satisfaction and productivity [42]. The ultimate objective of using social media in the workplace is to build social networks. This research reveals the positive effect of social media on the employee’s job performance with a mediating result of organizational structure. Based on these discussions, the following hypotheses of the study are proposed:

**H1:** *Work-related social media usage has a positive and significant impact on work performance.*

**H2:** *Social-related social media usage has a positive and significant impact on work performance.*

Extra-role behavior refers to all those activities that include some extra effort beyond the mere compliance or maintenance of the employees’ cooperation [43]. Extra-role behavior plays an essential role in the organization’s effectiveness to run the organization smoothly by affecting its performance. Proactive behavior, creativity, and knowledge sharing are critical extra job role behaviors exhibited by an employee in a workplace [44].

Proactive behavior is defined as the relatively stable tendency to affect and change the environment. People raised in an aggressive environment or one that has such characteristics are more disposed to alter their circumstances deliberately. Proactive people are actively oriented, searching for knowledge, exploring the environment, and somewhat anticipating the future instead of reacting to the situation [45]. Knowledge sharing is the term used to define the act of making knowledge available to the people within the organization. The process in which people exchange their expertise and generate new knowledge is known as knowledge sharing. Employees need to fully participate in knowledge sharing to make learning accessible throughout the organization [46]. Creating new ideas and fueling innovation in organizational products, services, processes, and procedures is known as creativity. Lastly, the capacity of the employee to positively react to change or unexpected conditions is known as adaptivity [47].

Organizations are increasingly empowering their employees where they are required to do more than their required formal job. The depiction of extra-role behavior increases organization productivity and effectiveness by helping transform innovation and adaptivity. These are voluntary behaviors that are not mentioned or specified in any employment contract; however, they are helpful and are an essential resource for organizational functionality and contribute to an increase in productivity [48].

Human behavior inside workplaces demands growing flexibility, making working roles, obligations, and responsibilities less rigid. Substantial literature on anticipated worker behaviors has led various researchers [49,50] to distinguish between in-role behaviors and actions that went beyond formal duties (extra-role behaviors). The authors of [51] defined extra-role behaviors as “work behaviors that are not always connected to work necessary tasks but contribute to the social and psychological elements of the organization,” such as “volunteering to carry out task activities that are not technically part of the job and aiding and working with others in the organization to get tasks performed” [52]. These extra-role actions are a hallmark of an organization’s responsiveness and flexibility when workers “engage in task-related behaviors at a degree that exceeds minimally necessary or commonly expected levels” [53]. Regarding the role and task fluidity owing to frequent change, and according to the cited literature [54,55], we thought it would be beneficial to assess the influence of social media usage on workers’ willingness to conduct non-job-related activities. When employees demonstrate extra-role behaviors and strong job engagement, managers perceive that they are extremely dedicated to the business as a whole [56]. Affordance theory, signaling theory, and a cognitive processing viewpoint all come together to help explain how these impressions may emerge. The affordance hypothesis [57], which is derived from ecological psychology, proposes that perception is formed from the interaction between an actor and his or her environment. An affordance, in particular, is a possibility or prerequisite for action [58].

**Figure 1:**
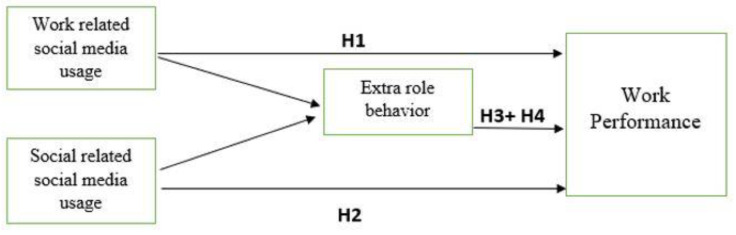
Conceptual Framework of the study.

In today’s organizational setting, managers, as actors, use environmental signals from employees as preconditions, or affordances, from which they may make future decisions. This idea is supported further by signaling theory [59] and the cognitive processing model. According to signaling theory, as managers do not have comprehensive knowledge about their workers, they rely on readily available and recognizable signals when making judgments about their employees’ attitudes and behaviors. Similarly, the cognitive processing model proposes that, due to restricted information processing capacities, individuals use shortcuts when forming attributions about the attitudes and behaviors of others [60]. We expect managers to utilize workers’ extra-role performance as a cue to infer employees’ commitment to the organization, which is consistent with affordance, signaling, and cognitive processing viewpoints. Moreover, performance should indicate to the management that an employee is effectively devoted (emotionally attached) and desires to remain with the business because he or she strongly connects with it [61]. The conceptual framework of the study is shown in Figure 1. Social media technologies have rapidly permeated workplaces, and businesses have intentionally implemented such tools to help their employees and improve their corporate operations [62]. We found it beneficial to assess the influence of extra-role behavior on workers’ motivation to perform similar tasks while using social media for both work- and social-related tasks at the workplace. Therefore, based on the above-mentioned discussions, the following hypothesis of the study is proposed:

**H3:** *Extra-role behavior mediates the relationship between work-related social media usage and work performance.*

**H4:** *Extra-role behavior mediates the relationship between social-related social media usage and work performance.*

## 2. Materials and Method

A quantitative research approach is used in this study. The study has been conducted in a non-contrived setting, and the unit of analysis is individual; the targeted respondents are addressed individually. This research aims to analyze the employee’s work performance through social media usage at the job place. The purpose of this research is to examine the responses of employees’ performance in a company to work- and social-related social media use, as well as their overall performance. In this study, the unit of analysis is the individual. The study’s target audience consists of organizational employees who perform duties at various levels of the corporate hierarchy (top, medium, and bottom) in both public and private corporations

### 2.1. Instrumentation

Primary data were gathered using an extensive and comprehensive questionnaire, which included questions on work-related and social-related social media use, as well as job performance. Respondents were also required to provide demographic information. A self-administered questionnaire was made available to participants both online and in person. Google forms were used to create an online questionnaire that was sent to participants because data collecting was a major difficulty during a worldwide epidemic, online questionnaires were used to gather information from participants. Since the present research was carried out during the COVID-19 pandemic, it was impossible to interact with employees who worked in an organization for the purpose of data collecting. A total of 500 questionnaires were distributed. Out of this total, 200 questionnaires were provided online and 100 were delivered in person, resulting in a total of 300 questionnaires being returned and completed. Replies from employees were inspected after data were collected, and 30 incomplete responses from an online survey and 19 incomplete responses from the physical survey were thrown away, leaving a total of 241 full responses after data were analyzed. As a result, the acceptance rate was nearly 48%. Furthermore, the minimal sample size for this research was determined by multiplying the number of items on the scale by “5–10 times” the number of items on the scale [63]. As a result, the total number of items in the research is 43, and the minimum sample size for the study will be 145 (43 × 5 = 215). Rascoe also proposed the rule of thumb, which states that, when the population of the research is unknown, an acceptable sample size of more than 30, but less than 500, should be taken into account [64].

The questionnaire was designed with close-ended questions to measure the constructs, which are the primary concern of this study. These questions were related to the demographic profile of the respondent. The current study used a questionnaire consisting of 43 items measuring four variables. Four items were used to measure work-related social media usage and four items of social media usage for the social purpose were adopted from the Voss (2003) study [65]. To measure the extra-role behavior 17 items by Claes et al. (2005), 13 items by Zhou and George (2001), 4 items by de Vries (2006), and 3 items by Griffin (2007) were used. The work performance of the employees was calculated by adopting 6 items from Kuvaas (2006) [66,67,68,69,70]. Likert scale was used, with the responses ranging from 1 = strongly agree to 5 = strongly disagree.

### 2.2. Ethical Statement and Participant Consent

Since this research project involves the participation of human participants, this research was given written permission after being reviewed by the University of Punjab in Lahore (ethical approval no. # IBIT/RC/202139). Participants in both the online and field surveys were required to sign a permission form, which stated that the information they provided would only be used for this research. The current study followed the ethical guidelines of the Helsinki Declaration. In addition, participants had the option to discontinue the data collection process at any time if they felt uncomfortable in revealing their information.

### 2.3. Data Analysis Tools and Techniques

The latest tools and techniques have been used in this research to answer the research questions. Statistical Package for the Social Sciences (SPSS 23 developed by IBM, Armonk, NY, USA) was used to carry out data cleansing, coding, and screening. The statistical package SPSS is also used for the analysis of demographic profiles and descriptive statistics. Partial least square Structural Equation Modelling was used for hypothesis testing and to analyze the structural and measurement model of the study. Both structural and measurement models have been analyzed with the help of PLS-SEM. Cronbach alpha, composite reliability, variance inflation factor (VIF), and the Heterotrait–Monotrait (HTMT) Ratio have been calculated to assess the measurement model. To evaluate the structural model, path coefficient, coefficient of determination (R square), and adjusted R square have been estimated.

## 3. Data Analysis and Results

### 3.1. Demographics

To investigate job-related and social-related social media use and work performance in a business, a multistage random sample approach was utilized. This technique helps to produce more genuine and clear data since it is more time efficient. All enterprises were divided into two main groups, namely, public and private, for this study. Six organizations were chosen at random from among all public and private organizations. The employees of these companies were divided into two groups: employees working in the HR department and all other department employees, including their supervisors. The personnel were further subdivided according to their position in the corporate structure, which included top-level, middle-level, and lower-level employees. Answers from corporate and governmental sector entities accounted for about 63.5 percent and 36.5 percent of total responses, respectively. Moreover, 61.3 percent of the final sample was comprised of non-HR employees, and 38.8 percent were made up of HR employees. Furthermore, the middle level of the corporate hierarchy has the largest proportion of responses (54.4 percent), while the lower level of the corporate hierarchy has the lowest percentage (17.5 percent). Table 1 represents the information related to the questionnaire distributed and collected from the organization.

Table 2 summarizes the demographic statistics of the respondents (*n* = 241) by summing all the demographics in the study along with their categories and the frequencies and percentages. There are 58.8% female and 41.5% male respondents in the research study. Moreover, 63% of participants are from private organizations, and 36% are from public sector organizations. The most dominant age group was between 20 and 25, representing 25.72% of respondents, and the minority age group was above 40. The most dominating group with the highest qualifications was the master’s, and the minority group was Ph.D. scholars. The most dominating group in monthly salary was below 25,000, and the minority group was between 75,000 and 100,000. The dominating group with total working experience was between 1 and 5 years, and the minority group was 10–15.

### 3.2. Measurement Model Assessment

In the first phase of the reflective measurement model assessment, internal consistency reliability is measured with the help of Cronbach’s alpha and composite reliability. Cronbach’s alpha and composite reliability value of the extra role behavior (ERB) have 0.789 and 0.797, respectively. Cronbach’s alpha and composite reliability values of the social-related social media usage (SRSMU), work performance (WP), and work-related social media usage (WRSMU) were above the critical level of consideration. Table 3 shows that all the reflective indicators of each of the constructs depict consistency in measuring that construct.

Heterotrait–Monotrait ratios (HTMT) were assessed to check the discriminant validity of the constructs. HTMT values that are less than 0.9 are considered critical for evaluating the construct’s discriminant validity. The relationship of ERB→WP has the highest HTMT value, i.e., 0.631, while WP→WRSMU has the lowest value, i.e., 0.326. Table 4 depicts that all the values are below the critical level of assessment, and, hence, it proved that discriminant validity is established.

Collinearity issues are checked with the help of the variance inflation factor (VIF). VIF should be above 0.20. Values that are below 0.20 are considered non-significant, and there are collinearity issues in the indicators. Values of VIF estimation range from 3.162 to 4.172. By evaluating VIF values, we can confirm that there are no collinearity issues in the indicators.

### 3.3. Structural Model Assessment

For testing the hypothesized relationship, path coefficient values and their significance are evaluated. According to Hair (2019), standard bootstrapping of 5000 samples was considered for evaluation [63]. Both t and *p* values were considered to determine the significance of the path coefficient of the model. The t values greater than 1.96 are considered significant for the study [71,72]. The path coefficient value of the relationship between work-related social media usage (WRSMU)→work performance (WP) is 0.223. The t statistics and *p*-value of the relationship WRSMU→WP are also significant at 3.126 (t > 1.96). Path coefficient values of the relationship between social-related social media usage (SRSMU)→work performance (WP) is 0.312. The t statistics and *p*-value of the relationship SRSMU→WP are also significant at 4.516 (t > 1.96). Table 5 represents the significance of the path coefficient. 

The coefficient of determination (R^2^ Value) of work performance (WP) is 0.527. It is considered a moderate impact as it is above 0.5. Similarly, the R^2^ value of extra-role behavior (ERB) is 0.632, and it is regarded as having a substantial impact on endogenous constructs because the R^2^ value is near 0.75 (substantial impact). Table 6 represents values of the coefficient of determination (R^2^ value). 

Path coefficient values of the relationship between work-related social media usage (WRSMU)→extra-role behavior (ERB)→work performance (WP) is 0.177. The t statistics and *p*-value of the relationship WRSMU→ERB→WP are also significant at 2.126 (t > 1.96). Similarly, Table 7 represents the path coefficient value of the relationship between social-related social media usage (SRSMU)→extra-role behavior (ERB)→work performance (WP), which is 0.300. 

**Figure 2:**
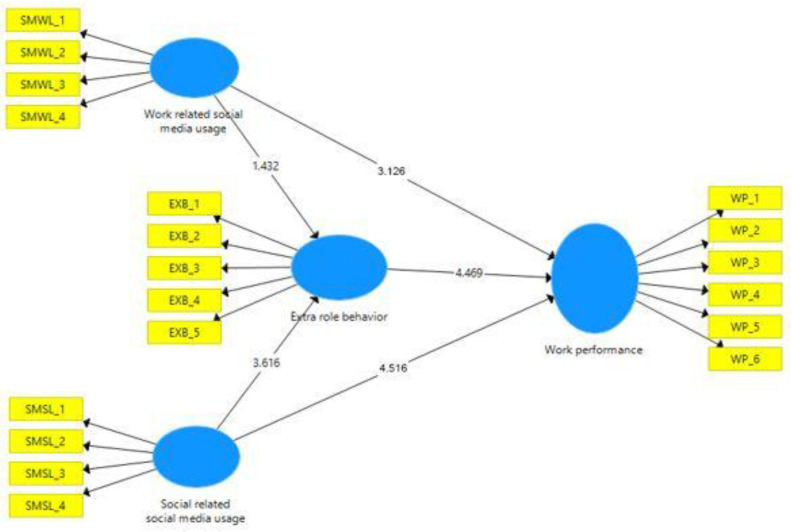
SEM model of the study.

Similarly, Figure 2 depicts the SEM model of the study. The t statistics and *p*-value of the relationship SRSMU→ERB→WP are also significant, i.e., 3.627 (t > 1.96).

## 4. Discussion

This study aims to explore the impact of social media usage for work-related and social-related purposes on the work performance of employees. The recent research depicted that self-motivation and self-efficacy and the use of social media have a significant and direct impact on the learning performance of employees. However, social media usage mediated the relationship between the social learning construct and learning performance [73]. Moreover, it has been shown that social media usage for a work-related purpose increases the efficiency of the employees and enhances creativity and problem-solving skills at the workplace [74].

Addiction to social media has increased in the workplace. The internet is turning into a significant source for online users to look for content or social gratification. One of the main factors that affect employees’ performance in the workplace is internet abuse and social media addiction. Research has explored the influence of personality factor-like locus of control and self-esteem, which significantly affect employee internet addiction [7].

Another study revealed that employees feel relaxed and work with more concentration when they are allowed to socialize and remain connected with friends and family at the workplace with the help of social media sites [75]. Similarly, another research study revealed that the role of social media supports the transactive memory system for increasing the knowledge creation capability and absorptive capacity of the employee, which in turn influences the team’s creative performance. Results show that, if the organization invests in social media, it will enhance the meta-knowledge within the team that will positively impact the team performance of the employees [76].

In their study, van Zoonen et al. (2014) highlighted that work-related social media serves in different ways, i.e., in information sharing, relationship management, and organizational ambassadorship. Employees enthusiastically use their social media accounts to contribute to the well-being of the organization. Similarly, another study revealed that employees could create network ties, shared vision, and trust by utilizing social media that facilitates the enhancement of their performance [11,77].

The advent of social media in many organizations empowers a new method of communication among employees. Another research study revealed that the hedonic and utilitarian values influence employees to use social media for their work irrespective of their gender, which helps increase their performance [78]. Enterprise social media affordances (visibility, association, edibility, and persistence) influence social media site ties (instrumental, expressive), which have a significant impact on the job and innovative job performance of the employee at a workplace [79]. From the above discussion and results of the data analysis, it is concluded that social media usage at the workplace, both for work-related and social-related purposes, enhances work performance, brings efficiency, and motivates employees to add more value to their daily tasks.

Extra-role behaviors are considered as the extra effort put in by the employees beyond their what their job roles and job descriptions demand. Another study revealed that self-efficacy and job satisfaction are leading factors in extra-effort job roles, and employees demonstrated extra-role behaviors [43,47]. Nowadays, organizations expect more from employees than their designated job roles. Knowledge sharing and helping others by going the extra mile are fundamental predictors of extra-role behavior [80]. In contrast, social media allows employees to share knowledge and enhance productivity. The current study concluded that extra-role behaviors mediate the relationship between social media usage (work and social purposes) and work performance.

## 5. Conclusions

While many studies have been conducted on social media usage in a particular context, studies investigating social media usage at the organization level are still small in number. Therefore, the current research analyzed the impact of both perspectives, i.e., work-related social media usage and social-related social media usage, on employees’ work performance at the organizational level. This study also explored the mediating effect of extra-role behaviors on work-related social media usage and social-related social media usage work performance relationship.

The first crucial key finding of the current study is that social media usage for both work and social purposes positively and significantly affects employees’ work performance. In other words, social media usage increases the work performance of employees working in different organizations. This study also highlighted that extra-role behavior significantly mediates the relationship between social media usage (work and social) and employees’ work performance. Extra-role behaviors successfully increase employees’ work performance when using social media for work and social purposes.

The use of social media in the workplace has improved employee performance. For example, the use of social media for work and for social purposes has a significant impact on enterprises’ cost reduction, innovativeness, and competitive advantage when it comes to marketing activities, procedures, and work. Making clear regulations about the use of social media at work also helps to boost the firm’s interactions with its customers and subsequently increases customer loyalty. In addition, it increases brand exposure, making it possible to reach a large number of consumers, and is easier for customers to find information about a company.

Because social media is a cheaper and quicker means to interact, exchange information, and innovate, businesses should encourage their staff to utilize it for their everyday tasks and marketing-related activities. Firms operating in developing nations should use social media because it provides a variety of new methods to conduct business, resulting in the development and maintenance of intellectual capital. This research highlighted the significance of social media and the consequences of using it.

## 6. Implication Limitations and Recommendations

This study has many theoretical and practical implications. The first theoretical implication of this study is that it contributes to the existing literature by providing a clear picture of social media usage for different purposes and its significant gain. Many studies have investigated the use of social media in a single context. However, there are two important reasons to use social media, i.e., for work and social purposes, therefore the current study explored the two perspectives of social media usage to investigate their impacts in organizations because they might lead to different outcomes.

Another essential contribution of the current study is the mediating effect of extra-role behaviors the on social media usage for work and social purpose–work performance relationship. Previous research studies have investigated the direct impact of social media usage on job satisfaction, organization engagement, and performance. However, along with direct association, the current study has also explored the effects of social media usage on work performance while considering extra-role behavior as a mediator. Moreover, it has been found that social media usage for work and social purposes increases the employees’ involvement in tasks which are not included in the formal job description and leads to better work performance of employees. This is an essential contribution as it demonstrates to organizations that fact that they can increase their employees’ work performance by involving employees in extra-role behavior such as helping others, contacting clients, and providing services to them through different social media usages. To access and examine the diverse behaviors and perceptions of people regarding the use of social media and its impact on the work performance and the mediating role of extra-role behaviors, data were collected from a sample population of employees working in the different sectors of a developing country.

The findings of this study provided some practical insights into organizations that, instead of just looking at the adverse effects of social media usage, also considered the beneficial aspects. Managers should not blindly ban social media usage. However, they should make strategies and rules that would define the acceptance of social media usage in the workplace, as social media usage engages employees in extra-role behaviors, which ultimately increase employees’ work performance. Organizations can take advantage of social media in different ways to promote themselves and their products. Organization departments might host brainstorming meetings or keep a blog with questions and answers to keep the dialogue going. A straightforward, unambiguous internet and social media policy is critical for expressing to workers the boundaries of appropriate online communication and behavior. It is important to educate employees and employers on why or how the social media policy is relevant to their day-to-day tasks. Managers should determine the most effective way for employees to utilize social media during work hours. It is important to remember that social media networks are a fantastic channel for growing a brand’s image and generating a devoted consumer base, and this opportunity should not be neglected.

The outcomes of the present study would be more generalizable and valid if the data had been gathered from employees working in developed and other underdeveloped countries. Results might be varied when moving across different cultures because social media usage varies in different countries. Another limitation of this study is that the sample is not representative of the whole population of employees in organizations. Therefore, the results cannot be generalized to all social media users in organizations. We have examined only two social media usage perspectives, i.e., work-related social media usage and social media usage, and these can be further subdivided to obtain more accurate and valid results in future studies.

The final limitation of the current study is that, as employees have self-reported their work performance, there might be a chance of overestimation because employees usually overestimate the extent of their work. Future studies should aim for a longitudinal study as the number of social media users increases over time; therefore, the impact of social media usage on work performance and whether it would be changed must be investigated. Future studies should dig deep into the concept of social media usage by exploring the impact of social media usage on some new variables other than job satisfaction, job commitment, and organizational commitment.

## Figures and Tables

**Table 1:** Number of questionnaires distributed and collected.

Organizations	Questionnaires Distributed	Questionnaires Collected	Private or Public Organization
Sapphire Textile Mills Limited (STML)	75	42	Private
Nishat Chunian GroupLimited	80	35	Private
Dalda Pvt Limited	85	44	Private
Descon Engineering Limited	70	32	Private
Wapda House Lahore Secretariat	80	38	Public
Punjab Provincial Cooperative Bank Limited (PPCBL)	110	50	Public
**Total**	241	500	

**Table 2:** Demographic distribution of the respondents.

Variable	Category	Distribution	
		Frequency	Percentage
**Gender**	Male	99	41.1
	Female	142	58.9
**Age**	20–25	62	25.72
	25–30	53	21.99
	30–35	56	23.23
	35–40	37	15.3
	Above 40	33	13.69
**Highest Qualification**	Less than B. A	13	6.6
	BS 2-years	40	16.5
	BS 4-years	41	17.01
	Masters	88	36.5
	MPhil	67	27.08
	Ph.D.	5	2.07
**Working sector**	Public	88	36.5
	Private	153	63.5
**Current organization work experience**	No experience	72	29.87
	1–5	90	37.3
	5–10	59	24.4
	10–15 above	20	8.29
**Monthly Salary**	Below 25,000	88	36.5
	25,000–50,000	58	24.0
	50,000–75,000	49	20.3
	75,000–100,000	19	7.88
	Above 100,000	27	11.2
**Total work experience**	No experience	50	20.7
	1–5	74	30.7
	5–10	76	31.5
	10–15 above	41	17.01

**Table 3:** Internal consistency reliability.

	Cronbach’s Alpha	Composite Reliability
ERB	0.789	0.797
SRSMU	0.834	0.831
WP	0.858	0.862
WRSMU	0.897	0.823

**Table 4:** Heterotrait–monotrait ratio.

	ERB	SRSMU	WP	WRSMU
ERB				
SRSMU	0.595			
WP	0.631	0.456		
WRSMU	0.372	0.418	0.326	

**Table 5:** Significance of path coefficient.

Suggested Paths	Original Sample (O)	Mean (M)	Standard Deviation (STDEV)	T Statistics	*p* Values
WRSMU→WP	0.223	0.202	0.025	3.126	0.000
SRSMU→WP	0.312	0.314	0.036	4.516	0.000
WRSMU→ERB→WP	0.177	0.179	0.029	2.126	0.000
SRSMU→ERB→WP	0.300	0.304	0.037	3.627	0.000

**Table 6:** Coefficient of determination (r^2^ value).

Endogenous Construct	R Square	R Square Adjusted
ERB	0.632	0.621
WP	0.527	0.518

**Table 7:** Summary of research questions, hypothesis, and significance of path coefficients.

Research Question	Research Hypothesis	Significance of Path Coefficient	Hypothesis Proved
Does work-related social media usage impact the employees’ work performance?	Work-related social media usage has a positive and significant impact on work performance	3.126 (t > 1.96), *p* = 0.000	Yes
Does personal use of social media impact the employees’ work performance?	Social-related social media usage has a positive and significant impact on work performance	4.516 (t > 1.96), *p* = 0.000	Yes
Do extra-role behaviors mediate the relationship between work-related social media usage and employees’ work performance?	Extra-role behavior mediates the relationship between work-related social media usage and work performance	2.126 (t > 1.96), *p* = 0.000	Yes
Do extra-role behaviors mediate the relationship between social-related social media usage and employees’ work performance?	Extra-role behavior mediates the relationship between social-related social media usage and work performance.	3.627 (t > 1.96), *p* = 0.000	Yes

## Data Availability

All relevant data are within the paper and according to the consent signed by the participants which stated that the information they provided would only be used for the purposes of the study.

## Abbreviations

ERB	Extra-role behavior
SRSMU	Social-related social media usage
WP	Work performance
WRSMU	Work-related social media usage
